# Biological lessons for strategic resistance management

**DOI:** 10.1111/eva.13616

**Published:** 2023-11-15

**Authors:** Daniel T. Blumstein, Norman A. Johnson, Nurit D. Katz, Samuel Kharpatin, Xochitl Ortiz‐Ross, Eliseo Parra, Amanda Reshke

**Affiliations:** ^1^ Department of Ecology and Evolutionary Biology University of California Los Angeles Los Angeles California USA; ^2^ Department of Biology University of Massachusetts Amherst Massachusetts USA

**Keywords:** microbial resistance, pesticide resistance, social change, social resistance, sustainability

## Abstract

Biological resistance to pesticides, vaccines, antibiotics, and chemotherapies creates huge costs to society, including extensive morbidity and mortality. We simultaneously face costly resistance to social changes, such as those required to resolve human–wildlife conflicts and conserve biodiversity and the biosphere. Viewing resistance as a force that impedes change from one state to another, we suggest that an analysis of biological resistance can provide unique and potentially testable insights into understanding resistance to social changes. We review key insights from managing biological resistance and develop a framework that identifies seven strategies to overcome resistance. We apply this framework to consider how it might be used to understand social resistance and generate potentially novel hypotheses that may be useful to both enhance the development of strategies to manage resistance and modulate change in socio‐ecological systems.

## 1 INTRODUCTION

Colloquial and formal definitions view resistance as an opposing or retarding force or substance. Often, resistance is viewed in the context of a change model, wherein resistance is a force against a change from State A to State B (e.g., Taplin & Clark, 2012). Resistance can be found at many levels of biological organization as well as in several social science disciplines, including psychology and sociology.

In venturing across fields, we need to be cognizant of the “jingle fallacy”, wherein the same term has different meanings. Here, the ubiquity of “resistance” as a term can create challenges in communication across disciplines and even within disciplines as it can be used differently in different fields and subfields. Notably, resistance is used in several different ways within biology. To avoid further confusion, we define biological resistance as a heritable property of the organism that reduces or eliminates harm from an agent (see Tabashnik et al., 2014 for further information). For example, a change in the efflux pump of a bacterium that reduces its susceptibility to antibiotics would be biological resistance. As a heritable trait, biological resistance can evolve via any of the evolutionary processes, including mutation, selection, and genetic drift. Biological resistance can arise via several mechanisms, including avoiding the harmful agent, sequestering the agent, destroying the agent, or rendering the agent inert.

Resistance appears in another and almost opposite form in landscape ecology/genetics. Here, landscape resistance describes how different landscapes impede or restrict the movement of organisms, across different surfaces or areas: landscape features with high resistance inhibit the movement of organisms while those with low resistance permit easy movement (e.g., Landguth et al., 2012; Spear et al., 2010; Zeller et al., 2012). Landscape resistance incorporates physiological costs involved with movement through the surface as well as reductions in survival and/or fecundity associated with such movement (Ghoddousi et al., 2021). Note that this landscape resistance is a property of the geographic feature but is also dependent on the interaction between the feature and the organism: a shallow stream may have a high resistance for a rodent but a low resistance for a bear. The concept of landscape resistance benefits the modeling of organisms and/or genes across geographic landscapes (Landguth et al., 2012). Human activity can increase or decrease landscape resistance depending on the circumstances: a road could decrease resistance by providing a corridor for the movement of an organism; conversely, a busy road could increase resistance by decreasing that organism's survival. This human‐induced landscape resistance is called anthropogenic resistance (Ghoddousi et al., 2021), which is structured by variation in the way risks are perceived.

Another type of resistance—wildlife resistance—is also used in biology. Reducing human–wildlife conflicts often requires interventions, such as the use of bear‐proof trash bins (Pienaar et al., 2015). Wildlife resistance is resistance to these interventions, and it can limit the utility of these interventions. Examples of how wildlife resistance could arise include habituation to various repellents (Blumstein, 2016) or behavioral innovations that render interventions ineffective (Barrett et al., 2019). For instance, raccoons (*Procyon lotor*) in Toronto, Canada (Doubek, 2018) and sulfur‐crested cockatoos (*Cacatua galerita*) in Sydney, Australia (Goodyear, 2021) have learned to open animal‐resistant bins. Non‐lethal hazing is often ineffective (Breck et al., 2017; Tidwell et al., 2021). Additionally, wildlife develops overall tolerance to human disturbance (as seen when animals living around humans permit humans to approach them closer than those in rural areas before fleeing; Samia et al., 2015), which can enhance their survival in urban areas but can also increase conflict through increased proximity to humans (Madden, 2004), and an increased perceived need for lethal control.

In human psychology, resistance generally refers to the resistance to change (Messer, 2002). Psychological resistance is often more of an inertia than an active force, but it can manifest as a more active form of resistance under some conditions (Jost, 2015). Advertisers, and now influencers, need to overcome that resistance to change habits. Similarly, governments and employers establish nudges (Thaler & Sunstein, 2008) to overcome psychological resistance and change behavior. Thaler and Sunstein's (2008) examples include having default contributions to employer‐sponsored retirement plans to overcome the resistance to people making a decision to save for their retirement and having organ donation be the default decision on a driver's license rather than having people opt‐in to organ donation to overcome the psychological resistance of donating organs after death.

Currently, we face a series of existential threats to civilization (Bradshaw et al., 2021) that require massive structural, societal, and behavioral changes. Sociological resistance to reducing CO_2_ in the atmosphere comes from fossil fuel companies that have massive sunk costs in an old system and will pay immediate costs of change (Supran & Oreskes, 2021), as well as, to a lesser degree, from individuals resistant to changing personal behavior (Palm et al., 2020). Chemical companies resist clean air and clean water laws that require them to pay an immediate compliance cost (Oreskes & Conway, 2010). Psychological resistance can limit our ability to prevent and manage disease. Although vaccines are an important global tool for disease management, there have been those who have resisted taking them since their invention (Berman, 2020).

Given the ubiquity of resistance across life and society, are there common insights that can be identified to better understand it? We identify seven general strategies to manage resistance (i.e., prevent its evolution/emergence) and find that they often require human behavioral change—whether applying integrated pest management (IPM)—which focuses on the ecosystem, adopts a long time window, and explicitly incorporates a variety of techniques that include biological control, habitat manipulation, modification of cultural practices, and the use of resistant varieties to manage agricultural pests (UCANR, 2022) to prevent overuse of pesticides, or changing medical or agricultural practices to avoid overprescribing antibiotics. Biological and psychological resistance intersect in these complex socio‐ecological systems (e.g., Jørgensen et al., 2020) because human psychological resistance to change can impact the successful use of strategies for managing biological resistance.

Attempting to apply lessons learned from the study of biological resistance to sociological and psychological resistance is a major challenge with a wide scope that will require much analysis from numerous groups. It cannot be fully addressed in a single paper. We regard our efforts as a first step designed to bring attention to the endeavor and generate productive discussions. Given our backgrounds and interests, we focus mostly on environmental matters but recognize that there are lessons to be learned for the application of public health measures (e.g., COVID‐19), as well as lessons that might help us better understand the dynamics of social resistance movements. Also importantly, while we focus on examples of preventing resistance, in some cases, we may wish to increase resistance (as seen when encouraging people to use crosswalks rather than jay‐walking or use trails in parks to avoid trampling vegetation). Insights developed to prevent the evolution of resistance may provide novel strategies to manage resistance in many contexts.

We also acknowledge the ethical and moral ramifications as these insights are applied to increasingly complex systems (from bacteria to wildlife to humans). We caution against ill‐informed application, particularly in more complex systems, and suggest that our lessons from biology be viewed as potentially testable hypotheses that may be applicable to managing resistance in socio‐ecological systems. And we acknowledge that this framework could be applied by public health officials and democratically elected politicians to reduce resistance to what might be perceived as positive social changes (encouraging people to be more physically active each day or to save more money for their retirement), as well as by industrial lobbyists and autocrats to increase resistance against changes (preventing the creation of a more resilient society that is less reliant on fossil fuels).

## 2 MANAGING BIOLOGICAL RESISTANCE

Various types of biological resistance (including resistance to antibiotics, pesticides, vaccines, and chemotherapy treatments) have enormous public health and welfare consequences. The evolution of antibiotic resistance remains a major public health threat; bacterial antimicrobial resistance alone killed an estimated 1.27 million people in 2019 (Murray et al., 2022). Cancer, which kills some 10 million people annually worldwide (Ferlay et al., 2021), remains a challenge to treat in part because of resistance: cancer cells within individuals receiving chemotherapy evolve resistance to the chemotherapy agents (Aktipis, 2020; Natterson‐Horowitz et al., 2023). The rapid evolution of resistance to antimalarials prevents effective treatment of malaria (Winegard, 2019), which killed at least 627,000 people in 2020 and sickened at least 241 million people annually (WHO, 2021). Lastly, insects and other organisms are responsible for the destruction of up to 40% of key global food crops (Savary et al., 2019). The effectiveness of pesticides is limited because of the evolution of resistant organisms. Managing such biological resistance is truly a grand challenge; yet, biologists have made some progress with various management strategies and measures.

Managing biological resistance requires some understanding of genetic and ecological factors that affect resistance. Resistance genotypes may arise de novo or may already be present in a population (Messer & Petrov, 2013). This distinction is important when considering which treatments/chemicals should be applied, but it is also relevant in determining the frequency of resistant individuals in a population. New mutations can be assumed to be rare when they first arise, but if resistance is already present, it may already be widespread. The higher the frequency of resistant individuals, the harder it will be to reduce the population size. To manage resistance, we must aim to reduce the frequency of resistant individuals. Broadly, this could be achieved in two ways: by reducing the total population size to the point where it cannot sustain positive growth (i.e., by creating an Allee effect; Stephens et al., 1999), or by reducing the fitness of resistant individuals (e.g., through increased immigration and competition with susceptible individuals that have increased fitness when there is no treatment). Note also that the fitness of resistant and susceptible genotypes may change over space and time (South et al., 2020), and also immigration, and more generally source–sink dynamics, can increase the frequency of mutations in a population and enhance resistance (Perron et al., 2007).

Genetic dominance is important for resistance outcomes. Consider the case where resistance is conferred by a single allele. When the resistant allele is dominant, both homozygous resistant and heterozygous individuals will be resistant to treatment. When the resistance allele is recessive, only the homozygous resistant individuals will be resistant to the treatment. Hence, resistance will be easier to manage when it is a recessive trait. Treatment dosage can determine the dominance of the resistant allele (Georghiou & Taylor, 1986). Under a high dosage, only homozygous resistant individuals are likely to survive (functionally recessive), while under a low dosage, both homozygous resistant and heterozygous individuals could survive (functionally dominant). Note that with either spatial or temporal dose gradients, dominance patterns may change, leading to “windows of dominance” that can affect the dynamics of resistance (see South et al., 2020, for details and references). Also note that single genes with pleiotropic effects can create costs that prevent the evolution of resistance to a specific environmental driver (Baucom, 2019; Johnson, 2022).

Resistant alleles are generally assumed to be mildly deleterious before treatment (Durão et al., 2018), so their initial frequency in a population is likely to be low. However, under persistent treatment, the fitness of resistant alleles will increase, and modifier genes will have enough time to integrate/move resistance alleles such that disadvantages are reduced or eliminated (which results in more “stable resistance”) (Georghiou & Taylor, 1986). Therefore, early intervention is critical. It is also important to note that selection for modifier genes may increase the rate of resistance evolution over time. Importantly, this may occur even if the treatment is not applied continuously. Trade‐offs may increase the cost of resistance (e.g., Basra et al., 2018), and a key question to answer is what are the conditions under which resistance is costly (Strauss et al., 2002) to evolve because costly mechanisms of resistance are likely to be selected against when a treatment is removed. By contrast, if resistance is not costly, it may persist following the cessation of a treatment. Compensatory evolution, wherein modifier alleles at other loci evolve to mitigate the negative effects of a resistance allele, can also reduce the cost of resistance (Baucom, 2019; Johnson, 2022).

Although most modeling efforts assume that resistance is due to allelic differences at a single locus, resistance can be polygenic (Hobbs et al., 2023). Hobbs et al. (2023) developed a quantitative genetic model to explore the efficacy of different insecticide resistance management strategies for polygenic traits and found that using high doses of two different insecticides was the best strategy examined to delay the evolution of resistance.

When choosing treatments, we must also consider cross‐resistance (Hobbs et al., 2023; Périchon & Courvalin, 2009). Resistance to one treatment may also confer resistance to other kinds of treatments (e.g., the *kdr* gene confers resistance to both DDT and pyrethroids because they both interfere with sodium gates along the axons of the nerve cells–Silver et al., 2018). Therefore, new treatments should target different mechanisms if we are to delay the evolution of resistance. The nature of the cross‐resistance may also affect the optimal treatment regime (see Hobbs et al., 2023, for more details).

In addition to (positive) cross‐resistance, there is also negative cross‐resistance wherein resistance to one treatment causes increased susceptibility to a second treatment (Pittendrigh et al., 2008). For example, a mutation in the *para* gene results in resistance to DDT but also causes hypersensitivity to deltmethrin in *Drosophila* flies (Pedra et al., 2004). A growing list of other genetically caused negative cross‐resistances in a variety of organisms has been documented (Pittendrigh et al., 2008). Negative cross‐resistance has the potential to be exploited in managing agricultural pests, such as by pyramiding two or more negative cross‐resisting toxins or by adding an agent to refugia that is negatively cross‐resistant to the primary management agent (Pittendrigh et al., 2008). To date, such ventures have not been well exploited in agricultural or insect vector pathogen settings; however, see Kurtak et al. (1987) for an example of how they have been applied in control of *Simulium damnosum*, the fly vector of the river blindness pathogen.

Negative cross‐resistance has been applied to clinical evolutionary medicine (Raymond, 2019). Chan et al. (2016, 2018) have shown that *Pseudomonas* bacteria that evolve to become resistant to an aquatic phage lose their resistance to many antibiotics and have used these phages to treat people with chronic *Pseudomonas* infections (see also Burmeister et al., 2020; Johnson, 2022).

An organism's ecology and life history are important determinants in the evolution of resistance. Organisms with shorter generation times will evolve genetic resistance at a faster rate. Similarly, greater fertility (or reproductive potential) should also confer greater resistance because they can “tolerate a higher intensity of selection” (Tabashnik & Johnson, 1999). Polyphagous insect pests and other generalists are also less likely to evolve resistance than specialists (like monophagous pests) because they are likely to have more untreated alternative resources they can use (Georghiou & Taylor, 1986). Similarly, a smaller proportion of the generalist population is likely to be exposed to the treatment. However, generalists, by virtue of being generalists, may also be more plastic and/or have more available mechanisms to develop resistance (Normark & Johnson, 2011). This may be important if all their resources are being affected, which we may see in wildlife and urbanization.

Migration can play a critical role in the evolution of resistance in two ways (Wang et al., 2022). When resistance is a recessive trait (as discussed above), the absence of migration would result in homozygous resistant individuals mating with other homozygous resistant individuals (because they are prevalent in the population), thereby producing more homozygous resistant offspring. However, with migration from or into an untreated population, resistant individuals are more likely to mate with homozygous susceptible individuals and produce heterozygous susceptible individuals. Migration also increases competition between resistant and susceptible individuals. In the absence of treatment (whether temporary or permanent), susceptible individuals are assumed to have a fitness advantage and are therefore likely to help reduce the growth of resistant populations.

The presence of susceptible individuals can be promoted by creating refugia—areas within a treatment patch that are not treated (Tabashnik et al., 2013). Some of these individuals will be in refugia during a treatment and therefore cannot evolve resistance to the treatment. There may be both spatial and temporal refugia. For instance, insects engaging in diapause are not eating and will not be susceptible to pesticides that require consumption. Refugia can be artificially created by excluding a segment of a population from treatment.

Resistance does not evolve at the same rate in all organisms (Baucom, 2019; Georghiou & Taylor, 1986; Johnson, 2022). Even within the same species, resistance may evolve more rapidly in some populations (e.g., Forgash, 1981). Moreover, some organisms may never evolve resistance (e.g., corn borers—*Ostrinia nubilalis*—failed to develop resistance to DDT despite extensive treatment). There are many factors that influence the evolution of resistance, but they can be broadly categorized as genetic, biological/ecological, and operational. The more ways there are to achieve resistance, the more likely it will evolve (Georghiou & Taylor, 1986).

### 2.1 Considerations for managing resistance

Given our understanding of the mechanisms by which biological resistance emerges, there are at least four initial considerations in managing resistance. First, *treatment selection* is essential. It's important to identify the treatments with the fewest resistance avenues and for which resistance comes at greater fitness costs (Baucom, 2019; Johnson, 2022; Wong et al., 2012). If multiple treatments are applied, treatments should target different mechanisms to avoid cross‐resistance, a concept known as degree of treatment heterogeneity and the likelihood that more than one pesticide is used against a set of resistance genes over some period of time (Bourguet et al., 2013; South et al., 2020). Ideally, one should choose treatments that foster negative cross‐resistance (resistance to one treatment confers greater susceptibility to the other—Beckie & Tardif, 2012). Second, the *timing* of treatments is essential. Early intervention is usually preferable, as treatments are generally more effective before resistance spreads (Comont et al., 2019; Mueller et al., 2005). Moreover, the deliberate timing of treatments through periodic application can be an effective strategy, as described in the section that follows. Third, the treatment *dosage* matters and, in many cases, should be kept high to avoid creating a functionally dominant resistance trait (Helps et al., 2017). Here, it is also important to consider treatment decay. If, after initial application, the treatment remains in the environment, this will be the equivalent of persistently treating the population at a lower dosage, which promotes resistance (e.g., South et al., 2020). Furthermore, persistently low dosages can also prevent immigration by killing immigrants. We also recognize that, in some circumstances, slowly increasing a dose can sometimes lead to the evolution of strong resistance (e.g., Bell & Collins, 2008; Perron et al., 2006). Fourth, *heterogeneity of treatments* is important (Baucom, 2019). And the degree of treatment heterogeneity refers to the likelihood that more than one pesticide is used against a set of resistance genes over some period of time (Bourguet et al., 2013).

### 2.2 Seven general strategies to manage resistance

From these four guidelines, we have identified seven strategies to prevent or delay the emergence of biological resistance. These strategies provide a framework for application.

1. When possible, *prevention* is the best strategy (Raymond, 2019). Adopting preventative public health strategies that reduce the spread of disease and avoiding the application of pesticides through ecological management or removing attractants are strategies to prevent the need for treatments that will select for resistance. This may not always be practical. The goals of public health and disease prevention can often conflict with resistance management. For example, in malaria control, insecticide‐treated bed‐nets and other treatments are often used with little regard for resistance management instead with the aim of reducing disease transmission. This also means that resistance management may often occur in the context of disease control. In some cases, drugs or pesticides are still used even when there is considerable resistance (e.g., artemisinin combination therapy for malaria, Watson et al., 2022), despite the insights garnered from the idyllic conditions of mathematical models. Moreover, when dealing with vector‐borne diseases, strategies to limit resistance may conflict with strategies to limit the spread of the pathogen (Sisterson, 2022).
2. Wildlife management provides an underappreciated mechanism to reduce the evolution of resistance by *rerouting or redirecting* organisms away from a desirable target (Smith et al., 2015). Redirecting animals away from contested resources, either by providing better options or by increasing the real or perceived costs of access, prevents the need to apply deterrents that are susceptible to the cultural evolution of resistance
3. An important strategy to overcome resistance is to increase the amplitude or force. In the context of controlling a cancer, a parasite, or a pathogen, resistance can be overcome by *increasing the dosage* of a treatment until all targets are eliminated. This strategy is more likely to work when applied early to a smaller population without widespread resistance. There is still a risk that resistance to high dosages will develop, and damage to non‐target organisms can result. When the necessary dosage is too costly or intolerably high, we must look for a different strategy or re‐evaluate our desired outcome.
4. Resistance often comes with costs, but costs have been shown to be variable and not inevitable in a wide range of biological systems (Johnson, 2022). In order to hinder the evolution of resistance, treatments that create the *largest costs of resistance* should be used.
5. The application of *multiple treatments* helps prevent the evolution of resistance to any single treatment. The choice of treatments becomes critical in preventing cross‐resistance. Previous reviews (Bourguet et al., 2013) have evaluated the effectiveness of four main multiple treatment strategies in preventing the evolution of resistance to drugs or pesticides by pathogens and pests. These strategies—responsive alternation, periodic application, mosaic treatment, and combination treatment—are detailed in Box 1.
6. *Adaptive therapy* works by a given treatment being used to maintain the population of the target below a certain threshold while preserving enough susceptible individuals to prevent the evolution of resistance. Adaptive therapy's (Gatenby et al., 2009) goal is not complete elimination but rather coexistence. A given treatment is used to maintain the population of the target below a certain threshold while preserving enough susceptible individuals. A critical threshold is defined above which the target population becomes unacceptably harmful and below which costs are tolerated or natural defenses and competition from other agents may control a target. Applied to cancer, chemotherapeutic agents shrink a tumor and then chemotherapy is stopped. Over time, the tumor grows, and another bout of chemotherapy is applied. Integrated pest management also follows the principles of adaptive therapy.
7. Adaptive therapy illustrates an example from a broader category of resistance management that we refer to as *redefining the goal*. Here, the goal is not to eliminate a target population but to reduce the costs to the organism or population. For example, it reframes the goal from “eliminating the cancer” to “reducing the likelihood that a cancer kills the patient”. By doing so, we have effectively reduced the likelihood of selecting more resistant targets.

BOX 1 **Types of multiple treatment strategies.**

Four main modes of multiple treatment strategies have been commonly used in attempts to thwart resistance. Here we briefly review these strategies.

**Responsive alternation** (Bourguet et al., 2013), also called sequence (Madgwick & Kanitz, 2023), is the most widely used multiple treatment strategy but is rarely effective. Responsive alternation relies on the repeated application of a single treatment until it is no longer effective (due to the widespread development of resistance), at which point a new treatment is applied. From a public health or agricultural standpoint, response alteration as a deliberate strategy should be employed cautiously, recognizing that it might be counterproductive, particularly if it is difficult or expensive to create new and effective chemotherapies.

**Periodic application** (Coyne, 1951), **also called rotation** (Madgwick & Kanitz, 2023), is similar to responsive alternation in that they both rely on a sequential (heterogenous across time) and uniform (homogenous across space) application of the treatment. However, in periodic applications, the goal is to switch between treatments before widespread resistance to a given treatment has evolved. Ideally, the switch would occur within a generation timeframe to maximize efficiency. Periodic treatments that target different mechanisms will be more successful at preventing the evolution of resistance than periodic applications of treatments that target the same mechanism.

Unlike the previous strategies, a **mosaic treatment strategy** (Madgwick & Kanitz, 2023; Muir, 1977), also known as mixture (Raymond, 2019), focuses on applying different treatments in different patches, such that a portion of the population receives treatment A while another portion of the population receives treatment B. This strategy prevents the spread of resistance to any single treatment in the general population because patches ensure that, at a population level, susceptibility remains to other treatments. In other words, mosaic treatments rely on creating heterogenous selection pressures that should hinder the evolution of resistance (Raymond, 2019). To maximize its efficiency, patches should be smaller than the dispersal distance of individuals because it relies on the free movement of individuals from one treatment patch to another. Mosaic treatments are usually most effective when resistance is low (Raymond, 2019).

While mosaic and periodic application have similar levels of success, a **combination approach (also called mixture;** Madgwick & Kanitz, 2023), which entails the simultaneous and uniform application of multiple treatments, has been shown to be the most efficient (Bourguet et al., 2013; Madgwick & Kanitz, 2022, 2023; Mani, 1989). The goal is to eliminate the target by “hitting it hard.” Individuals resistant to one agent are likely to be killed by the others. Moreover, the combined use kills more individuals in total (see Madgwick & Kanitz, 2023 for more detail). This strategy assumes that most individuals will not already have resistance to *both* treatments. It is essential to get the doses correct and to ensure that they are synergistic rather than redundant in terms of the mechanisms targeted.

## 3 MANAGING SOCIOLOGICAL AND PSYCHOLOGICAL RESISTANCE

Given these seven biologically‐derived strategies, in addition to problems where resistance is mostly a problem of biological evolution, we now consider two additional types of general resistance problems: (1) those where resistance is behavioral or cultural, and (2) those that involve more complex socio‐ecological or bio‐social systems. The context of a problem may constrain the application of some strategies and require careful consideration. Given that the strategies emerged from considering biological evolution and are being actively applied there, we focus below on the other two types of problems, illustrate how these insights might be applied, and view our suggestions as hypotheses that are ripe for testing (Table 1).

**TABLE 1:** Examples of how resistance management strategies could be applied or are being applied in different contexts.

Strategy	Biological examples	Behavioral or socio‐ecological examples
(1) Prevention	Planting diverse crops and rotating them as opposed to planting monocrops may help prevent specialist predators that must be subsequently controlled	Human‐wildlife conflicts with bears can be prevented by not building homes in bear habitat and by developing and enforcing regulations that prevent people from feeding bears directly or indirectly (by using non‐bear‐proof trash cans or by planting fruit trees)
(2) Reroute or redirect	No good examples	Increasing the perceived predation risk or the cost of accessing a desired resource can be used to devalue this resource and redirect animals to other places. Electric fences can help reduce crop foraging by elephants
(3) Increase amplitude or force	In the context of controlling a cancer, a parasite, or a pathogen, resistance can be overcome by increasing the dosage of a treatment until all targets are eliminated. This strategy is more likely to work when applied early to a smaller population without widespread resistance	Creating strong disincentives for doctors to provide antibiotics without tests that specify which antibiotic may work. Or banning the use of antibiotics on otherwise healthy farm animals
(4) Use treatments that create the largest cost of resistance	If the cost of resistance is known, select an antibiotic with the greatest cost of resistance	No good examples
(5) Use multiple simultaneous treatments	See Box 1	To address vaccine hesitancy, provide more information about vaccine safety, provide payments for vaccination, and have vaccine mandates required for work or entry
(6) Use adaptive therapy	Chemotherapeutic agents shrink a tumor, and then chemotherapy is stopped. Over time, the tumor grows, and another bout of chemotherapy is applied. Integrated pest management also follows the principles of adaptive therapy	Driving bans when air pollution is particularly bad are an example of adaptive therapy being applied to reduce air pollution in cities
(7) Redefine the goal	The goal of treating cancer is to have the patient live, not necessarily eliminate all metastatic cells. In some sense, both adaptive therapy and integrative pest management do just this	Tolerating some wildlife crop foraging or depredation may be more practical and sustainable than trying to eliminate it all

### 3.1 Behavioral and cultural resistance

There are many examples of efforts to influence human behavior and social norms to achieve an outcome, such as reducing lawns in xeric areas or the use of private cars in many locations. The success of changing social norms may be improved by applying lessons we have identified from managing the evolution of resistance. We recognize the complexity of human systems and the potential ethical issues raised when applying lessons from biological systems to cultural systems. Nevertheless, we consider how the lessons from biology may potentially help us understand human change.

Consider the compelling need to change human behavior to reduce the likelihood of climate‐driven biological and societal collapse (Bradshaw et al., 2021). Models that assume that humans, once educated about the facts of climate change, will voluntarily change their behavior to reduce the impact of climate change have had limited success (Palm et al., 2020). Why? Because humans resist changing their social norms both actively (e.g., the immediate trigger of the French yellow vest uprising in 2019 followed an increase in fuel taxes) and passively (people may not change their consumption patterns after the introduction of a new policy because they are simply not inspired or sufficiently motivated to change). What might the application of strategies to create the needed cultural shift in how we use fossil fuels look like?

Humans are behaviorally heterogeneous, and viewing this variability in the context of resistance suggests that insights from combination therapy may be valuable. Consider COVID vaccination reticence. There are a number of different reasons why people fail to get vaccinated (Solís Arce et al., 2021). For some, providing more information about safety will eliminate hesitancy. For others, providing incentives (payment for vaccination) is effective, while others will respond better to disincentives (vaccine mandates required for work or entry). Thus, we expect that the most effective social‐change interventions will include some form of combination treatment (see Bourguet et al., 2013), whereby several strategies are employed concurrently. The scale at which individuals receive treatments is also important. For example, an individual may be exposed to several combinations of targeted social information types (combination treatment), but overall exposure to population treatments is in waves (periodic treatment).

Challenges to behavioral change are seen with wildlife management ‘treatments’ to address human–wildlife conflict, such as hazing and other techniques to repel ‘problem’ animals. These often fail because animals habituate to these treatments—they learn that non‐lethal deterrents are either not real threats or the cost is not sufficiently large, and their response to them declines (Schakner & Blumstein, 2016). Preventing such behavioral resistance motivates much wildlife biology. Many wildlife management tools inevitably lead to resistance. By focusing on different approaches, such as rerouting or redirecting organisms, they may prove more successful. Setting aside land for wildlife and not permitting human encroachment may be an effective way to reduce the need for additional resistance‐prone strategies (e.g., Mekonen, 2020), as is eliminating attractive foods that may draw bears and elk into backyards. Adopting a behavioral approach whereby the challenge is to attract and repel animals in ways that do not generate resistance to an explicit treatment can be a powerful way to view many wildlife management issues (Greggor et al., 2020).

Resistance to new costly social policies may be generally expected, and the uptake of beneficial strategies may be insufficient to create needed change. A mosaic strategy could apply beneficial strategies or treatments and apply them in one area (e.g., free public transportation in areas where people are not using public transportation) and apply another strategy in another area (e.g., large rebates for buying an electric car). Influencers could be important mechanisms (Johnstone & Lindh, 2018) by which information about policies would travel between locations and create a desire for the community to change behavior and/or support the adoption of new policies. Of course, influencers could also be counterproductive to public health and welfare, as seen with the recent rise of antivaccine messaging during the COVID‐19 pandemic (Durmaz & Hengirmen, 2022).

An example of rerouting might be applied to the problem of reducing CO_2_ emissions associated with personal travel. Instead of promoting electric vehicles, rerouting would provide free public transportation to reduce the need for individual cars. On the other hand, when Tesla built highly desirable and expensive luxury all‐electric cars that remained desirable without tax incentives, they redefined the goal—to buy electric vehicles because they were luxurious, not because they were environmentally friendly.

Adaptive therapy works by a given treatment being used to maintain the population of the target below a certain threshold while preserving enough susceptible individuals to prevent the evolution of resistance. While adaptive therapy might not generate new ideas to address climate change, it has been used to reduce air pollution. Bans on driving that are explicitly linked to air pollution levels have been used in some cities to reduce pollution (Rivera, 2021).

Reputational costs and benefits associated with politicians expressing their opinion may be a key to understanding many cliodynamic models of change (Turchin et al., 2017). Making it less reputationally costly for leaders and institutions to express or change specific opinions may reduce political resistance. For instance, it was the US Republican Nixon administration that passed, with bipartisan support, some of the most important environmental legislation in US history—the Clean Water Act, Clean Air Act, and Endangered Species Act (Brinkley, 2022). Americans from both political parties protesting for the environment and against pollution may have enabled that leadership because popular protests reduce the reputational costs of politicians changing their mind.

We should generally be wary of cross‐resistance. While speculative, this may be particularly relevant when dealing with taxes to reduce demand and may quickly lead to enhanced overall resistance against other taxes and thus be counterproductive. For instance, in France, in 2019, it is possible that relatively high taxes and a general feeling of inequity in taxation may have reduced the threshold to protest following a modest increase in fuel taxes.

### 3.2 Bio‐cultural resistance

Some important resistance problems require cultural change, yet their success has explicit biological consequences. Such bio‐social resistance problems are well illustrated by antibiotic resistance, a broadly recognized worldwide threat that is making formerly treatable infections untreatable. Importantly, addressing antibiotic resistance requires addressing issues at different scales. At the individual level, better hygiene may reduce the likelihood of contracting an infection, and individuals should be educated about the risks of inappropriate use of antibiotics. At the community level, many farmers overuse antibiotics preemptively to increase farm animal density. Similarly, clinicians overprescribe antibiotics, sometimes because of patient demands. At the national level, we lack regulations to prevent the misuse of antibiotics.

Scale matters in managing resistance, as seen with antibiotics, because resistance operates at the global scale. Thus, policies that may delay the evolution of resistance in one location (e.g., in some European countries, doctors do not routinely provide antibiotics without tests that specify which antibiotic may work best, and giving antibiotics to otherwise healthy farm animals is prohibited—Fair & Tor, 2014) may be rendered ineffective because resistant strains spread from countries without effective regulations. Importantly, changing the behavior of farmers and doctors generates its own cultural resistance. At this point, we recognize that there are general strategies to address resistance that could be applied locally, but these must be better modeled and studied in bio‐cultural systems. True success may require global coordination.

## 4 CONCLUSIONS AND LIMITATIONS

Applying the lessons learned from studies of managing biological resistance to managing psychological and sociological resistance requires sufficient similarity in the nature of both types of resistance. Biological resistance does not involve cognition, while cognitive reasoning can factor into psychological and sociological resistance. Some resistance is based on rational reasons; others are irrational. These differences are important to be aware of, but do they mean lessons from biology cannot be applied successfully?

In some cases, we know the genetic transmission patterns of resistance to antibiotics and other forms of biological resistance. We also know a lot about cultural transmission. However, we have not viewed the cultural transmission dynamics of psychological and sociological resistance in a similar way as we have biological resistance. Doing so would let us evaluate the similarity of patterns and processes that underlie it, and this is essential if we are to apply it to social resistance.

Adopting an interdisciplinary approach is essential to overcome broad resistance. Future work should address the following outstanding questions. We must better understand the situations when resistance is costly to better predict when it persists after selection is removed. We must better identify the effective treatment type, timing, and intensity for a given state of resistance. We must develop guidelines to identify the circumstances in which it is useful, valid, and ethical to apply biological strategies to social resistance. We must develop quantitative models to understand human habituation and cross‐resistance to social change messages. We must determine whether there are unique insights from cultural resistance that may help improve the development of resistance‐proof chemotherapies. We must clarify the interactions between biological, behavioral, and cultural forms of resistance.

While we have highlighted a number of ways that biological insights create lessons for managing cultural change, we also recognize that there may be insights from the social sciences that can create novel strategies to help overcome biological resistance. We hope this essay stimulates further interdisciplinary scholarship that helps us develop novel resistance‐proof therapies as well as more effective strategies to overcome the social resistance that plagues our attempts to create a more sustainable world.

## CONFLICT OF INTEREST STATEMENT

The authors declare that there is no conflict of interest.

## Data Availability

Data sharing is not applicable to this article as no datasets were generated or analyzed during the current study.
